# Writing and reading in the electronic health record: an entirely new world

**DOI:** 10.3402/meo.v18i0.18634

**Published:** 2013-02-05

**Authors:** Heeyoung Han, Lauri Lopp

**Affiliations:** 1 Department of Medical Education, Southern Illinois University School of Medicine, IL, USA; 2 Department of Family & Community Medicine, Southern Illinois University School of Medicine, IL, USA

**Keywords:** electronic health record (EHR), electronic medical record (EMR), reading, documentation, navigation

## Abstract

**Background:**

Electronic health records (EHRs) are structured, distributed documentation systems that differ from paper charts. These systems require skills not traditionally used to navigate a paper chart and to produce a written clinic note. Despite these differences, little attention has been given to physicians’ electronic health record (EHR)-writing and -reading competence.

**Purposes:**

This study aims to investigate physicians’ self-assessed competence to document and to read EHR notes; writing and reading preferences in an EHR; and demographic characteristics associated with their perceived EHR ability and preference.

**Methods:**

Fourteen 5-point Likert scale items, based on EHR system characteristics and a literature review, were developed to measure EHR-writing and -reading competence and preference. Physicians in the midwest region of the United States were invited via e-mail to complete the survey online from February to April 2011. Factor analysis and reliability testing were conducted to provide validity and reliability of the instrument. Correlation and regression analysis were conducted to pursue answers to the research questions.

**Results:**

Ninety-one physicians (12.5%), from general and specialty fields, working in inpatient and outpatient settings, participated in the survey. Despite over 3 years of EHR experience, respondents perceived themselves to be incompetent in EHR writing and reading (Mean = 2.74, SD = 0.76). They preferred to read succinct, narrative notes in EHR systems. However, physicians with higher perceived EHR-writing and -reading competence had less preference toward reading succinct (*r*= − 0.33, *p*<0.001) and narrative (*r*= − 0.36, *p*<0.001) EHR notes than physicians with lower perceived EHR competence. Physicians’ perceived EHR-writing and - reading competence was strongly related to their EHR navigation skills (*r*=0.55, *p*<0.0001).

**Conclusions:**

Writing and reading EHR documentation is different for physicians. Maximizing navigation skills can optimize non-linear EHR writing and reading. Pedagogical questions remain related to how physicians and medical students are able to retrieve correct information effectively and to understand thought patterns in collectively lengthier and sometimes fragmented EHR chart notes.

## Introduction

Electronic health record (EHR) systems are being nationally adopted with the expectation and evidence of improved health care quality in the United States (1–3). Even though physicians have a positive perspective about EHR systems, their ‘full function’ adoption is still low: 3.6% in 2006, 3.8% in 2007 (4), 4% in 2008 (5), and 6.9% in 2009 (6). Literature indicates that technical barriers and inappropriate design elements explain the slow adoption, (5, 7) and yet these issues are challenging to address. EHR studies between 1995 and 2005 are generally categorized into four topics: the presence of an EHR, EHR adoption, EHR functionalities, and EHR disparities (8). Since then, new EHR challenges have been discussed with a focus on workflow, interoperability, communication (9), the educational use of an EHR, (10–13) and organizational support and quality (14, 15). However, little attention has been given to the topic of physicians’ EHR usage regarding the aspects of writing and reading EHR-generated documentation.

Given the importance of quality chart notes for good medical practice and the different documentation structures of EHRs, it is imperative to investigate the different aspects of physicians’ EHR writing, reading, and navigation abilities. In traditional handwritten chart notes, all patient information, including chief complaint, medical history, physical exam, medications, diagnostic information, and assessment and plan are written down on paper. These paper notes are often created in a narrative format designed by a linear information structure for writers and readers. A narrative note is essentially a story containing a sequence of events or experiences. In a linear information structure, such as a paper document, information is created and presented in one place as an ordered collection of events.

Contrary to paper charts, EHRs are computer-based documentation systems. Literature shows that computer-based document systems include both structured documentation entry and free text processing (16). Structured data entry system can be useful for data reuse and analysis, but it requires a high learning curve for users. On the other hand, free text entry system, including dictation, allows flexibility and expressivity, but these are not efficient for data reuse and workflow. Rosenbloom et al. (16) discussed that each approach has its own benefit, and will continue to evolve.

An EHR contains structured information from varied sources that can be fragmented and not occur as an ordered collection of events (i.e., hyperlinked) (17). Literature in the field of human–computer interface found that it takes more time to find correct information in distributed and hyperlinked information systems that are not organized well than those in a linear format (18). Consequently, it may not be easy to identify critical clinical information in a timely manner using an EHR. In fact, a recent study reported that data-retrieving errors in an EHR are common in fourth year medical students even with 1 year of EHR experience (19). Failing to retrieve critical information can lead to poor documentation performance and health care practice. However, EHR notes can contain detailed information, such as vitals, medications, lab results, diagnostic reports, and prior structured documentation, in easier ways than in hand-written notes. These data come from different contributors adding to the notes as well as from lab and hospital interfaces, yet can be efficiently accessed with well-designed user interface. Zheng et al. (20) investigated internal medicine residents’ navigation patterns in an EHR and found three most frequently used paired patterns: ‘assessment/plan and diagnoses’, ‘order and medication’, and ‘order and laboratory test’. As they discussed, users’ information retrieval patterns and paths can be different from system developers’ intentions, so it is imperative to incorporate EHR usage patterns in designing user interface for data retrieval.

Using an EHR involves three general tasks: writing, reading, and system navigation. In traditional training settings, as Boulet et al. (21) noted, students are taught to write a chart note on paper placing an emphasis on accuracy, thoroughness, organization, sound interpretation, and logical coherence (21, 22). Writing concise, high quality chart notes has been a core skill in medical education (22). A succinct note is a summary of developments, expressed with brevity, which is valued in medical practice because it is more readable than lengthy discourse. The quality characteristics of a paper chart note are still valuable in EHR documentation. According to a recent study, approximately 64% of US medical education programs in 2009 allowed medical students to use EHRs (13). The primary use was for reading with a subset using EHRs for documentation as well. Considering the increased EHR adoption since then, (23) it is plausible that the trend has been expanded to other medical schools. To address this EHR trend in medical schools, a multidisciplinary group of clinical education recently provided a guideline regarding medical students’ EHR documentation (12). Their guidelines include a gradual exposure to templates, opportunity to practice order entry in the EHR, introduction of decision aids, and communication skills with a patient-centered approach.

However, while significant training efforts have been placed on creating chart notes, much less emphasis has been placed on reading them. In addition, there has been little emphasis placed on teaching navigation skills in EHR systems (17). According to Yudkowsky et al. (19), information-retrieving errors frequently occur in EHRs; therefore, the construct of EHR-reading competence is likely more challenging than paper chart note reading. Given that writing, reading, and navigating in an EHR system is substantially different and based on structured, hyperlinked, and distributed information, it is necessary to revisit the constructs of writing and reading in EHRs and to explore additional educational avenues.

Furthermore, as EHR systems replace traditional paper charts, a new method of writing and reading using digital templates and encounter forms is replacing the traditional handwritten method. A simple linear translation of the existing pedagogy into an EHR without careful reinterpretation of its meaning may cause poorer performance in writing and reading EHR chart notes. This can then lead to eventual poor healthcare practice.

Consequently, given these technological changes, more specific instructional guidelines focusing on digital writing and reading skills are needed. While meeting these needs is imperative, little empirical study exploring this challenge in medical education and clinical practice has occurred.

The threefold purpose of this study was to 1) determine physicians’ perceived competence in writing and reading EHR notes, 2) determine physicians’ preferences for the same, and 3) identify factors associated with perceived competence and preferences.

## Methods

The study was exempt from Institutional Review Board review. We developed a web-based survey instrument to measure physicians’ perceived EHR-writing and -reading competence and preference. The participants of the study were physicians who were using EHR systems in their practice at hospital(s) and an outpatient teaching institution located in the midwest region of the United States. Purposive and convenience sampling was used because those appropriate for the study were using EHRs for several years. In addition, they were invited based on relative ease of access. Physicians from general and specialty fields, working in inpatient and outpatient settings, were invited to the survey. Most of the physicians participating in the study worked at three different facilities simultaneously. We sent Human Resources (HR) staff in three hospitals an invitation email, including a link to the survey. Then, HR staff forwarded the invitation email to the physicians on behalf of the researcher. Two additional reminder emails were sent by each HR staff. Given that most physicians worked at three hospitals, they received up to six reminder emails. The total number of the physicians was obtained through HR data from each hospital. No monetary benefits were provided to the participants. No identification information was collected for anonymity and their response data were kept confidential.

Data collection was conducted over 2 months from February to April 2011. Quantitative data analysis, including descriptive statistics, factor analysis, reliability analysis, correlations, and regression analysis, was conducted using SPSS (PASW Statistics 18). To avoid type I error by multiple correlations, Bonferroni's correction (24) was considered to be conservative in interpreting correlations. However, Bonferroni's correction may also lead to type II error to reject significant correlations (25). Considering both aspects, in the current study, correlations above *r =*0.30, *p <*0.005 were interpreted as significant. Moreover, multiple regressions were used to find significant relationships without the risk of type I error. Non-EHR users and outliers were excluded from data analysis.

### EHR usage survey instrument

Based on literature review in chart note writing and EHR documentation functionalities, five categories were created: EHR-writing competence (21), EHR-reading competence (19, 21), and EHR-writing preference in terms of input format (typing vs. check boxes) (16, 26), EHR-reading preference in terms of output format (narrative vs. bullet points) (27), and EHR-reading preference (succinct vs. lengthy) (28). EHR-writing competence includes an ability to create thorough and complete notes, using an appropriate form in a timely manner. EHR-reading competence indicates an ability to retrieve and accurately interpret structured information stored in EHRs. EHR-writing preference indicates writers’ preference in data entry between structured documentation and flexible, free-text documentation. EHR-reading preference includes readers’ preference in information presentation in terms of narrative versus bullet points and succinct versus lengthy notes.

Fourteen five-point Likert scale items were created to constitute the five categories based on ease and usefulness of a technology acceptance model (29). An additional 12 questions were added for demographic information, EHR navigation skills, general computer skills, and EHR training priority in medical schools. The questionnaire was reviewed by a 10-member advisory group consisting of four physicians, a professor in medical education, a professor in psychology, a professor in computer science, one EHR educational specialist, one clinical assessment director, and one research assistant. The items were revised based on their feedback.

Factor analysis and Cronbach's alpha reliability test were conducted to provide validity and reliability of the instrument. Principal components and factor analysis yielded four distinct components with eigenvalues greater than 1 (see Table 1), which indicates that the instrument nicely distinguished the different measurement constructs. The EHR usage scale with the 14 items showed high reliability:

1. EHR competence in writing and reading (items 1–8, *α*=0.840)
2. EHR-reading preference in narrative (over bullet points) (items 9–10, *α*=0.928)
3. EHR-reading preference in succinct notes (over lengthy notes) (items 11–12, *α*=0.914)
4. EHR-writing preference in typing (over check boxes) (items 13–14, *α*=0.698)

**Table 1:** Factor loading of the survey items

		Component			
		
Categories	Items	1	2	3	4
EHR-writing competence	I could easily create a thorough patient chart note in the EHR.	0.76			
	EHR forms are helpful in completing the chart notes thoroughly.	0.67			
	*I sometimes feel that I create an incomplete note in the EHR.	0.68			
	*It takes too much time for me to enter information in the EHR.	0.60			
	*It is difficult to find an appropriate form for documenting in the EHR.	0.70			
					
EHR-reading competence	*It takes too much time for me to find information in the EHR.	0.73			
	*I often find patient chart notes created by other physicians in the EHRs are very difficult to understand.	0.59			
	When I read EHR chart notes, I can easily understand how the diagnosis assessment and plan were determined.	0.58			
					
Reading preference in narrative	Narrative chart notes are easier to understand than chart notes using bullet points.		0.90		
	Narrative chart notes are more beneficial to readers than chart notes using bullet points.		0.90		
					
Reading preference in succinct notes	Succinct chart notes are easier to understand than lengthy notes in EHR systems.			0.92	
	Succinct chart notes are more useful than lengthy notes in EHR systems.			0.91	
					
Writing reference in typing	I usually type in the text box rather than use check boxes in EHR forms to create patient chart notes.				0.89
	I prefer typing to using check boxes in EHRs.				0.83

Scales: 5 = Strongly agree, 4 = Agree, 3 = Neutral, 2 = Disagree, 1 = Strongly disagree

* Reverse coding.

## Results

Ninety-one physicians out of approximately 730 physicians (12.5%), from general and specialty fields, working in inpatient and outpatient settings, completed the survey. The initial response was from 58 physicians. After several reminders, it increased to 95. One non-EHR user, two incomplete responses, and one outlier were excluded from data analysis. Despite multiple reminders, this online survey response rate was low, but typical (30). Of interest, physicians’ response rate to an online survey is even lower than other populations (30, 31). Given that EHR use was not mandatory at the time of this survey, physicians who were not using EHRs did not participate in the survey. Consequently, the response rate among actual EHR users may be higher.

Participants had a mean age of 49 years (ranging from 28 to 69; SD = 10.02), were 58% male, were associated with 1–3 clinics, utilized one or two different EHR systems in their practice, and had a mean EHR use experience of 39 months. The majority reported comfort in navigating their current EHR systems (Mean = 3.54, SD = 1.07), good typing skills (Mean = 3.55, SD = 1.32), and competence in navigating computer software in general (M = 3.20, SD = 0.82, note that only this item was on a 4-point scale).

While the physicians had used EHR systems for over 3 years, they perceived their EHR-writing and -reading competence to be somewhat low (Mean = 2.74, SD = 0.76). Their perceived low EHR competence was not different between the two major EHR systems that they were using (*F*=0.23, *p =*0.88). There was no significant correlation between their perceived EHR competence and the number of EHR systems that they were using. The physicians found it difficult to find appropriate forms for documentation and felt they sometimes create incomplete EHR chart notes. When they read EHR chart notes, many found other physicians’ diagnosis and plan of care notes difficult to understand. They had high preference in reading narrative (Mean = 3.87, SD = 1.09) and succinct notes (Mean = 3.64, SD = 1.16). There was no preference found regarding the use of typing or using check boxes to document (Mean = 3.01, SD = 0.99). While the physicians preferred reading narrative and succinct notes, physicians with higher perceived EHR-writing and -reading competence had less preference for reading succinct EHR notes than physicians with lower perceived EHR-writing and -reading competence (*r*= − 0.33, *p <*0.001, see Table 2). In addition, physicians with higher perceived EHR-writing and -reading competence had less preference in reading narrative EHR notes than physicians with lower EHR competence (*r*= − 0.36, *p <*0.001).

**Table 2:** Spearman correlations

	EHR-reading preference in succinct notes	EHR-reading preference in narrative	Navigating skills in EHRs	EHR experience (month)	Medical specialty – surgery	Age	Gender (M1F2)	Typing skills	General computer navigating skills
EHR writing and reading competence	−0.33**(*n*=90)	−0.36** (*n*=90)	0.55** (*n*=90)						
EHR-writing preference in typing								0.29** (*n*=88)	
EHR-reading preference in succinct notes		0.24*(*n*=90)			0.25* (*n*=90)				
EHR-reading preference in narrative						0.22* (*n*=85)	−0.21* (*n*=89)		−0.26* (*n*=90)
Navigation skills in EHRs							0.26* (*n*=89)	0.32** (*n*=90)	0.28** (*n*=90)
EHR experience (Month)						−0.34** (*n*=79)			
EHR-training needs			−0.50** (*n*=88)	−0.23* (*n*=80)				−0.30** (*n*=88)	
Age							−0.30** (*n*=84)	−0.28** (*n*=85)	−0.30** (*n*=85)
Gender								0.40** (*n*=89)	
Typing skills									0.32** (*n*=90)

* Correlation is significant at the 0.05 level (2-tailed).

** Correlation is significant at the 0.01 level (2-tailed).

Physicians with higher navigation skills in EHR systems were more competent in reading and writing EHR notes (*r*=0.55, *p <*0.0001). Given that EHR navigation skills are correlated with typing skills, gender, and general computer navigation skills, regression analysis was conducted to identify the variable that contributed the most in predicting physicians’ EHR navigation skills. The regression model was significant with *R* ^2^ *=*0.19 (*F*=6.4*, p <*0.001). The significant independent variable was general computer navigation skills (*t*=2.88, *p <*0.005) while gender and typing skills did not significantly contribute to the model. This finding is consistent with the previous literature that navigation behavior is related to general computer skills (32).

While a recent study reported that older physicians who see more patients with more documented problems use novel EHR functionality, (33) the current study found that older physicians had experienced EHR systems for a shorter period of time than younger physicians (*r*= − 0.34, *p <*0.002). It shows that younger physicians have used EHR systems in their practice for a relatively longer time than older physicians. In addition, younger physicians’ general computer navigation skills were higher than older physicians. This may imply that younger physicians’ EHR adoption may have been higher than older physicians because of the breadth and length of exposure to technology.

Given that perceived EHR-training needs had negative correlations with EHR navigation skills, the length of EHR experience, and typing skills, regression analysis was conducted to identify the relative importance of the three variables associated with physicians’ EHR-training needs. The regression model result was *R* ^2^=0.35 (F = 14.6, *p <*0.001). The length of EHR experience (*t*= − 2.5, *p <*0.02) and the EHR navigation skills (*t*= − 5.1, *p < 0*.001) were found to significantly contribute to the model. This indicates physicians with higher EHR navigation skills would not want more EHR training. In addition, physicians who have used an EHR for a long time do not feel they need more EHR training. Neither physicians’ EHR writing and reading competence nor EHR navigation skills had positive correlation with their length of EHR experience. More than half of the physicians indicated that navigation in EHRs (59%), writing notes thoroughly (58%), and use of a computer during a patient encounter (56%) should be a priority in medical school EHR training.

## Discussion

The current study showed that while physicians have used EHR systems for over 3 years, they still had low perceived EHR writing and reading competence. Improving the physicians’ competence for writing and reading EHR chart notes becomes a critical issue for achieving the goal of improved health care using EHRs. For medical educators, the study findings provide pedagogical implications for chart note writing and reading in EHR systems.

Considering the findings reported above, it is plausible that EHR navigation training should precede general EHR training. There has been no specific EHR training guideline focused on EHR navigation skills in medical education literature. Physicians should be able to navigate and retrieve the right information in an EHR before creating and reading in the medical record. Without EHR system navigation skills, physicians may take more time to write and read patient EHR chart notes than they would on paper. Given the complex non-linear structure of EHR documentation systems, it is important to form a spatial cognitive map of an EHR system using strong spatial navigation skills (17, 34). The inability to navigate well or develop a cognitive map of an EHR system can limit the physicians’ ability to find relevant templates and information. For this reason, they would prefer reading narrative notes summarized in one location rather than reading a fragmented note that contained information from different locations in the EHR. For improved efficiency, it is recommended that physicians have navigation training opportunities to create a spatial cognitive map for an EHR.

Medical educators are faced with pedagogical questions related to how physicians and medical students are able to retrieve correct information effectively and to understand thought patterns in fragmented yet collectively lengthier EHR chart notes. Physicians often prefer to read narrative, succinct notes presumably because it is efficient to read the narrative. However, EHR notes are different and more difficult to write, read, and understand than handwritten paper chart notes (35). Electronic notes tend to be lengthier than handwritten notes (28). When the varied data, such as vitals, medications, lab results, diagnostic reports, and consultations, come together, they can make a collectively lengthier note than the traditional one. Due to the automaticity of many of the sections being included, notes quickly lose their succinctness. It will be important to prepare medical students to be able to retrieve and read collectively lengthier information in EHRs during their clinical practice training.

For those with higher perceived EHR reading and writing competence, the current study revealed that they preferred bulleted EHR notes to narrative notes than those with lower EHR-reading and -writing competence. This proves a natural phenomenon that with more system exposure, they are able to find the data they need in fragmented notes more easily. Reading patient information that populates an EHR chart note is usually neither linear nor narrative.

Due to physicians’ preference for succinct notes in general, focus on how to achieve and design succinct EHR notes must be encouraged in users who are new or have low EHR-reading and -writing competence. Regardless of the EHR competency level, all physicians feel frustrated while trying to navigate through a poorly organized, data-heavy EHR note. Formatting can be customized to place importance on certain sections of an EHR note. For example, an assessment, plan, and diagnostic justification can be formatted to occur at the top of a note to allow for easy communication of essential information (20). For these reasons, it is very important that physicians are involved in decisions that drive the formatting of an EHR note. They need to be involved in generating notes that contain essential information and are easily readable.

While perceived EHR competence did not improve with time of EHR use, physicians’ perceived training needs for EHR decreased. More structured EHR training can work for those clinicians new to EHRs, with a focus on strengthening their EHR system navigation skills. For those who have used an EHR for a long time, just-in-time learning strategies or job-aid type tips can be useful to help develop EHR competence rather than formal EHR training. This type of training is likely more useful to users that have already received initial formal training and have used the system.

This study has several limitations. First, the findings are based on physicians’ perception. Their EHR-writing and -reading competence, preference, and navigation skills were decided by their self-assessment. Actual measurement of their competence and skills should be investigated in future studies. Second, the small number of responses may result in the potential issue of type II error. The small sample size may not be sufficient to detect statistical difference; therefore, it is recommended to have more subjects in the future study. Finally, the physicians in the midwest were invited to participate in the study, so this selected population may not represent the physicians in the other areas. Physicians in rural clinics or varying geographic and demographic regions may provide different findings.

In conclusion, as the health care technology environment changes, medical education's pedagogical and technological approaches need to be revisited to meet the new environmental needs. Society requires physicians to be able to read, write, and work efficiently and effectively in EHR systems. EHR competence requires integrated digital writing and reading skills, which also extends to documenting workflows in distributed information systems (36, 37). Medical educators should help physicians and medical students develop EHR navigation skills to quickly find critical patient information and to understand thought patterns in fragmented, non-linear, yet collectively lengthier EHR chart notes. Without these EHR competencies, physicians will have difficulty in creating and accessing critical information that is necessary for making strong and accurate clinical decisions.
